# M2 macrophage-derived exosomes promote angiogenesis and improve cardiac function after myocardial infarction

**DOI:** 10.1186/s13062-024-00485-y

**Published:** 2024-06-06

**Authors:** Hongzhou Guo, Zeya Li, Bin Xiao, Rongchong Huang

**Affiliations:** 1 Department of Cardiology, Beijing Friendship Hospital, Capital Medical University, 95 Yong’an Road, Beijing, 100050 P. R. China; 2 Department of Cardiology, The Second Hospital of Dalian Medical University, Dalian, China

**Keywords:** M2 macrophages, Exosomes, Angiogenesis, Myocardial infarction

## Abstract

**Background:**

Myocardial infarction (MI) is a major cause of mortality and morbidity worldwide. The intercellular communication in post-infarction angiogenesis remains unclear.

**Methods:**

In this study, we explored the role and mechanism of action of M2 macrophage-derived exosomes (M2-exos) in angiogenesis after MI. M2-exos were harvested and injected intramyocardially at the onset of MI. Two distinct endothelial cells (ECs) were cultured with M2-exos to explore the direct effects on angiogenesis.

**Results:**

We showed that M2-exos improved cardiac function, reduced infarct size, and enhanced angiogenesis after MI. Moreover, M2-exos promoted angiogenesis in vitro; the molecules loaded in the vesicles were responsible for its proangiogenic effects. We further validated that higher abundance of miR-132-3p in M2-exos, which recapitulate their functions, was required for the cardioprotective effects exerted by M2-exos. Mechanistically, miR-132-3p carried by M2-exos down-regulate the expression of *THBS1* through direct binding to its 3´UTR and the proangiogenic effects of miR-132-3p were largely reversed by *THBS1* overexpression.

**Conclusion:**

Our findings demonstrate that M2-exos promote angiogenesis after MI by transporting miR-132-3p to ECs, and by binding to *THBS1* mRNA directly and negatively regulating its expression. These findings highlight the role of M2-exos in cardiac repair and provide novel mechanistic understanding of intercellular communication in post-infarction angiogenesis.

**Supplementary Information:**

The online version contains supplementary material available at 10.1186/s13062-024-00485-y.

## Introduction

Myocardial infarction (MI), one of the leading causes of mortality worldwide, occurs when the coronary artery is blocked by rupture or erosion of an atherosclerotic plaque, resulting in cell death in the ischemic and hypoxic territory [1]. Although timely interventional and pharmacological therapies have minimized myocardial injury and enhanced the survival rate of patients with MI, irreversible cardiomyocyte loss in the hypoperfused area and the development of adverse left ventricular remodeling lead to an appreciable proportion of heart failure or sudden cardiac death in several survivors [2, 3]. Therefore, additional effective therapeutic strategies are needed to improve the prognosis of patients with MI.

Accumulating evidence indicates that angiogenesis plays a critical role in cardiac repair after MI; the formation of new blood vessels salvages cells from death and attenuates adverse remodeling [4]. Angiogenesis after MI involves communication among the different cell types in the heart, and macrophages have emerged as the main driving factor. An intercellular interaction network in infarcted hearts constructed by single-cell RNA sequencing and a map of ligand-receptor pairs revealed that macrophages displayed the largest number of outbound connections, while endothelial cells (ECs) exhibited the largest number of inbound connections [5]. However, the roles of different macrophage subsets are distinct in cardiac repair after MI. M1 macrophages generate pro-inflammatory cytokines and clear cell debris, while M2 macrophages create anti-inflammatory cytokines, chemokines, and growth factors to promote cardiac repair [6, 7]. Hence, as shown by current studies, the timely transition of M1 macrophages to M2 macrophages is beneficial for alleviation of myocardial injury [8–10]. Similarly, the angiogenic properties of M1 and M2 macrophages differ; M2 macrophages produce higher levels of angiogenic growth factors and cytokines than M1 macrophages [11]. Meanwhile, M2 macrophages enhance tube formation, but M1 macrophages display the opposite effect in vitro [12]. Therefore, elucidating the mechanisms underlying the cardioprotective effects of M2 macrophages through angiogenesis may pave the way for a novel therapeutic target in MI.

In general, intercellular interactions can occur directly between adjacent cells via gap junctions, or indirectly over long distances through proteins and extracellular vesicles, including exosomes [13]. Exosomes mediate cross-talk between cells under physiological and pathological conditions by transferring signaling molecules, including functional proteins, mRNA, and microRNAs (miRNAs) [14]. Recently, studies have focused on the biological activities of exosomes as they have effects similar to their donor cells without the complexities of cell transplantation. However, the role of M2 macrophage-derived exosomes (M2-exos) in angiogenesis after MI has never been described. We hypothesized that M2-exos exert cardioprotective effects; we then established that M2-exos promote angiogenesis and improve cardiac function by transporting miR-132-3p to ECs. Our findings may lay the foundation for potential novel therapeutic approaches in MI.

## Methods

### Cell culture and conditioned medium

Human coronary artery endothelial cells (HCAECs), human artery endothelial cells (HAECs), human umbilical vein endothelial cells (HUVECs), and endothelial cell medium (ECM) were obtained from ScienCell Research Laboratories (Carlsbad, CA, USA). These primary ECs were cultured in ECM with 5% fetal bovine serum (FBS) and 1% endothelial cell growth supplement. THP-1 cells were derived from the Cell Bank of the Chinese Academy of Sciences, cultured in RPMI 1640 medium with 10% FBS and 100 ng/ml phorbol 12-myristate 13-acetate (Sigma-Aldrich, St. Louis, MO, USA) for 24 h (M0 macrophages). Next, macrophages were plated with 20 ng/ml IL-4 (PeproTech Inc., Rocky Hill, NJ, USA) for 48 h for M2 macrophage polarization or treated with 100ng/ml LPS (Sigma-Aldrich) plus 20ng/ml IFN-γ (PeproTech Inc., Rocky Hill, NJ, USA) for 48 h for M1 macrophage polarization. All cells were maintained in a humidified atmosphere of 5% CO_2_ at 37 °C and the media were changed every 2 days.

### Exosome extraction and protein quantification

Macrophages were cultured in RPMI 1640 medium supplemented with 10% exosome-free FBS for 2 days. Conditioned medium was collected and centrifuged at 2500 g for 30 min and 8500 g for 30 min to remove cell debris. Then, the supernatant was centrifuged at 120 000 g for 70 min twice, the pellet was washed with PBS, and centrifuged for another 70 min at same speed at 4 °C. Finally, the pellet was dissolved in PBS and passed through a 0.22 μm filter. Pierce BCA Protein Assay Kit was used to measure the protein concentrations of exosomes by means of a standard curve at an absorbance of 562 nm.

### Exosome characterization

Exosomes were identified and characterized by Nanoparticle Tracking Analysis (NTA), transmission electron microscopy (TEM), and western blotting. After dilution of exosomes in an appropriate volume of PBS, the size and concentration of exosomes were determined by a NanoSight analysis system (ZetaView, Particle Metrix 110, Germany), and the morphology and size of exosomes were observed under TEM (Hitachi, Tokyo, Japan). Additionally, exosome-related protein biomarkers were identified by western blotting.

### Exosome internalization assay

PKH67 solution (Sigma-Aldrich, MI, USA) was used to label exosomes according to the manufacturer’s protocols. Exosomes were incubated with PKH67 at room temperature for 4 min. Then, exosome-free FBS was used to terminate the reaction, and the exosome suspensions were centrifuged at 120 000 g for 70 min at 4 °C and resuspended in ECM. The PKH67 pre-labeled exosomes were incubated with ECs for 12 h, after which they were fixed in 4% paraformaldehyde, and stained with phalloidin and DAPI at room temperature and viewed with a confocal microscope (Olympus, Tokyo, Japan).

### Exosome mild digestion assay

Exosome suspensions were treated with proteinase K (TIANGEN, Beijing, China) at 37 °C for 30 min, followed by RNase (TIANGEN, Beijing, China) at 37 °C for 15 min to separate potential exosome-free RNAs and proteins. Alternatively, 0.1% Triton X-100 was used to pretreat exosome suspensions to destroy membrane structures and then combined with RNase and proteinase K to digest all RNAs and proteins.

### Cell viability assay

ECs were incubated with exosomes (3 µg/ml) or transfected for 48 h, then, cells were resuspended at a density of 800 cells/well in 96-well plates. At time points of 0 and 48 h, 20 µL of MTS solution (Promega, WI, USA) was added to each well and incubated for another 2 h at 37 °C. The absorbance of each well was measured at 562 nm by a microplate reader.

### Cell proliferation assay

EC proliferation was assessed through EdU proliferation assays (RiboBioInc, Guangzhou, China). 2.5 × 10^4^ ECs were treated with exosomes (3 µg/ml) in 24-well plates for 48 h. An aliquot of 50 µM EdU was incubated with ECs for 2 h and then fixed in 4% paraformaldehyde for 30 min. We used 0.5% Triton X-100 to pretreat ECs, followed by staining with Apollo and Hoechst, according to the manufacturer’s protocols.

### Cell migration assay

The migration ability of ECs was evaluated using Transwell assay (Corning, NC, USA). After incubated with exosomes (3 µg/ml) or transfected for 48 h, 2.5 × 10^4^ ECs were resuspended and seeded in the upper chambers in 0.5 ml serum-free medium, whereas the bottom chambers were filled with 0.8 ml medium containing 10% exosome-free FBS. After 12 h of migration, ECs were fixed and stained with anhydrous methanol containing crystal violet. The chambers were washed and the cells inside the chamber were wiped off. The cells were quantified in at least three random fields under a microscope (Olympus, Tokyo, Japan).

### Tube formation assay

The angiogenic ability of ECs was assessed using Matrigel tube formation assays. After incubated with exosomes (3 µg/ml) or transfected for 48 h, ECs were resuspended at a density of 1 × 10^4^ in 96-well plates with 70 µL Matrigel matrix (Corning, NC, USA) per well. After incubation at 37 °C for 6 h, the tube formation was captured by inverted microscopy and calculated using ImageJ software.

### Cell transfection

Oligonucleotides for miR-132-3p mimics and inhibitors, as well as their negative controls were obtained from GenePharma (Shanghai, China). Plasmids encoding *THBS1* were derived from Youbio (Changsha, China); the *THBS1* cDNA fragments were inserted into the pIRES2-EGFP vector. SiTran 2.0 (OriGene Technologies, Beijing, China) was used for transfection, according to the manufacturer’s protocols, and the transfection efficiency was tested after 48 h.

### RNA isolation and RT-qPCR

Total RNA of exosomes was extracted using the miRNeasy® Kit (Qiagen, Hilden, Germany). TRIzol reagent (Thermo Fisher, Waltham, MA, USA) was used to isolate RNA of cultured cells, according to the manufacturer’s protocol. RNA was quantified and cDNA was obtained from 500 ng of RNA using a reverse transcriptase kit (TakaRa, Tokyo, Japan), and qPCR was performed using Fast SYBR Green Master Mix (Thermo Fisher, CA, USA). Relative changes in mRNA and miRNA were quantified using GAPDH or U6 as the reference using the 2^−ΔΔCt^ method. The primer sequences are listed in the Supplementary material online (Table S1).

### Western blot analysis

Total proteins of cells or exosomes were obtained and the concentration was measured using BCA protein assay kit (Thermo Fisher, Waltham, MA, USA). Proteins of equal amounts were subjected to sodium dodecyl sulfate–polyacrylamide gel electrophoresis and then transferred to a polyvinylidene fluoride membrane (Millipore, MA, USA), which was blocked in 5% fat-free milk at room temperature for 2 h. The membranes were incubated with primary antibodies overnight at 4 °C and specific secondary antibodies for 2 h. Images of protein bands were captured using ChemiDoc XRS + system (Bio-Rad, CA, USA). The primary antibodies were as follows: anti-CD63 (1:1000, Abcam), anti-Alix (1:2000, ProteinTech), anti-TSG101 (1:8000, ProteinTech), anti-GM130 (1:2000, ProteinTech), anti-THBS1 (1:500, Santa), anti-ARID1A (1:1000, Abcam), anti-SPRED1 (1:1000, Abcam), anti-PTEN (1:2000, ProteinTech), anti-GAPDH (1:5000, PtroteinTech).

### Dual-luciferase reporter assay

The wild type or mutant 3´untranslated region (3´UTR) segment of *THBS1* was inserted into the pmirGLO vector. SiTran 2.0 was used for co-transfection of miR-132-3p mimics and *THBS1* 3´UTR or mutant *THBS1* 3´UTR. After 48 h of co-transfection, the firefly and renilla luciferase activity was determined by the dual-luciferase reporter assay system (Promega, WI, USA), according to the manufacturer’s protocols.

### Biotinylated miRNA pull-down assay

Biotinylated miR-132-3p mimics and negative controls were obtained from GenePharma (Shanghai, China) and transfected into ECs for 48 h. Lysis buffer containing protease and RNase inhibitors were used to lyse cells, followed by NaCl treatment to 1 M concentration. Streptavidin magnetic beads (Thermo Fisher, Waltham, MA, USA) were mixed with cell lysates at 4 °C for 2 h after blocking. Subsequently, the beads were rinsed with washing buffer, and then RNA was extracted using miRNeasy® Kit (Qiagen, Hilden, Germany) and analyzed using RT-qPCR, as previously described.

### Mouse MI model and exosome delivery

Eight to 10-week-old C57BL/6 male mice were purchased form Vital River Laboratory Animal Technology Co., Ltd (Beijing, China) and bred in a specific pathogen-free facility with ad libitum access to sterile food under staff supervision. All mice experiments were conducted in accordance with the Beijing Friendship Hospital Guide for laboratory Animals and approved by Institutional Animal Care and Use Committee of Beijing Friendship Hospital.

All mice surgeries and statistical analyses were conducted in a blinded fashion for intervention. The left anterior descending coronary artery was ligated to induce a MI model, in accordance with our previous report [15] and verified by ST-segment elevation in ECG and loss of color at the apex of the heart. A total of 20 µg of exosomes was delivered through intramyocardial injections at three sites around the border zone (10 µL each) at the onset of MI using a 30-gauge Hamilton syringe.

### Transthoracic echocardiography

Mice were anesthetized with 5% isoflurane inhalation and maintained at 2% isoflurane inhalation on a heating plate. A high-frequency ultrasound system Vevo® 2100 (VisualSonics, Canada) was used to evaluate the cardiac function of mice on day 28, post-infarction. M-mode tracings in parasternal left ventricular short-axis and long-axis were used to measure left ventricular wall thickness and left ventricular inner diameter at the end systole and diastole. The left ventricular ejection fraction (LVEF) and fractional shortening (FS) were subsequently determined.

### Vessel density quantification

CD31 immunofluorescence staining was used to quantify the vessel density in the border zone of infarcted hearts. In brief, the heart tissue sections were dewaxed and incubated with antibodies against CD31 (1:4000, Abcam) overnight at 4 °C, followed by incubation with specific secondary antibodies for 2 h at room temperature. At least five high-power fields were observed and positively stained cells calculated in the border zone of each section.

### Histological analysis

Mice were sacrificed 28 days after MI and triphenyltetrazolium chloride (TTC) staining and Masson trichrome staining were performed. The heart was perfused and sliced by transverse sectioning with a pre-cooled blade, followed by incubation with 1% TTC (Sigma-Aldrich) solution at 37 °C for 30 min to identify the infarcted areas. Masson trichrome staining was performed according to the manufacturer’s protocols (Sigma-Aldrich).

### Statistical analysis

Data were analyzed with GraphPad Prism 8 software and presented as mean ± SEM. Student’s t-test was used to analyze the differences between two groups and multiple comparisons were analyzed by one-way ANOVA. *P* < 0.05 was considered statistically significant.

## Results

### M2-Exos enhanced angiogenesis and cardiac function following MI

**Fig. 1:**
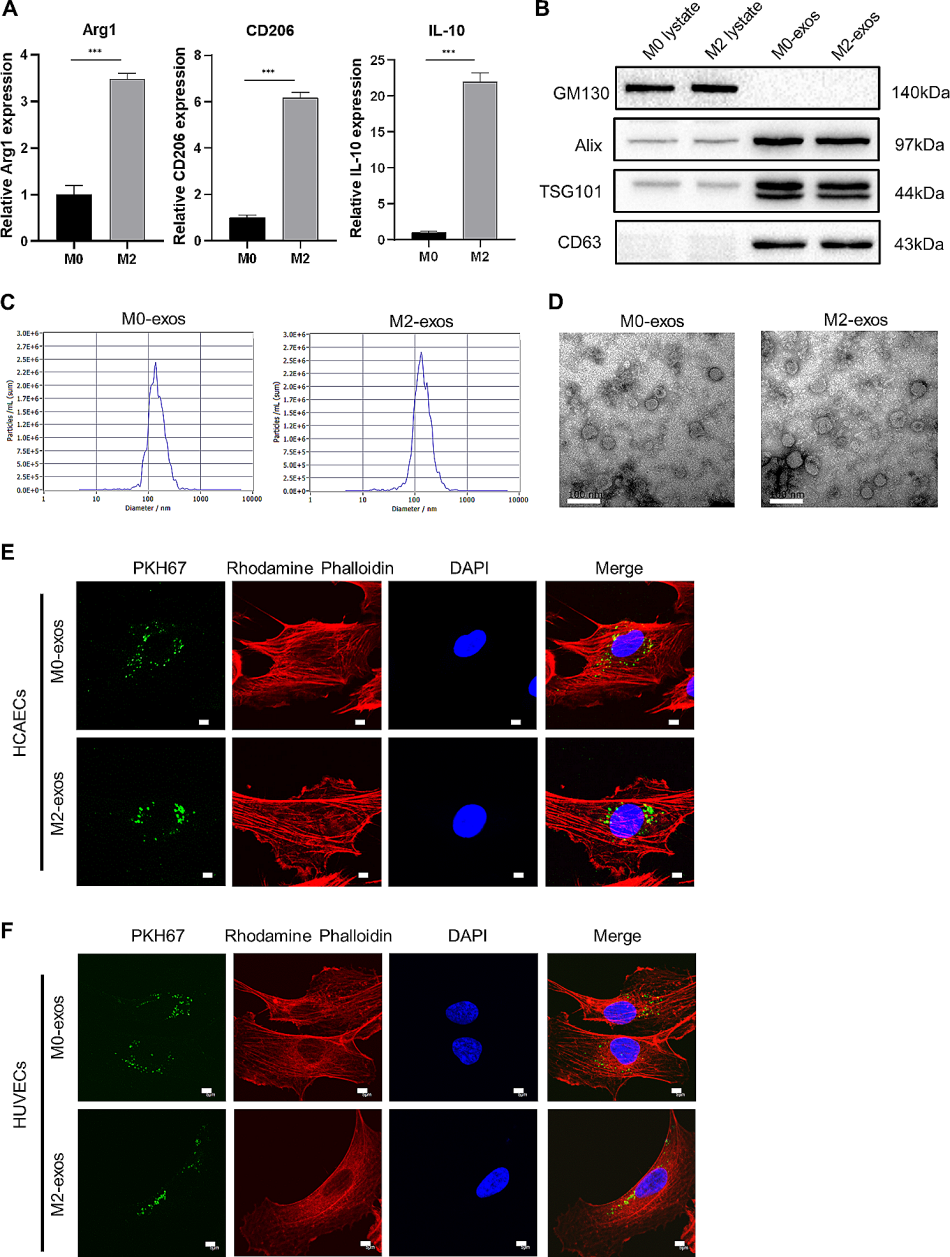
Identification of macrophages and their exosomes. (**A**) RT-PCR was used to measure the expression of IL-4 induced-M2 macrophage biomarkers, viz., Arg1, CD206, and IL-10, *n* = 3. (**B**) Western blot analysis was used to identify the expression of protein biomarkers of exosomes, TSG101, Alix, and CD63, and cytoplasmic biomarker GM130. (**C**) NTA was used to determine the concentration and size of the isolated exosomes. (**D**) Morphological images of exosomes were observed under TEM, scale bar = 100 nm. (**E**) PKH67-labeled exosomes were internalized by HCAECs, scale bar = 5 μm. (**F**) PKH67-labeled exosomes were internalized by HUVECs, scale bar = 5 μm. (Data are presented as mean ± SD; ***P < 0.001.)

THP-1 cells were added to PMA to induce macrophage formation, and then treated with IL-4 for conversion to M2 macrophages. The mRNA expression of M2 macrophage markers, such as Arg1, CD206, and IL-10 was confirmed by RT-qPCR (Fig. 1A). Then, HAECs were cultured in exosome-free conditioned medium (CM) of M2 macrophages and we found that the tube formation ability of HAECs was attenuated in the absence of exosomes (Supplementary material online, Figure S1). These results indicate that M2-exos may play a significant role in mediating angiogenesis. To clarify the effects and mechanism of action of M2-exos, we used ultracentrifugation to isolate exosomes from M0 and M2 CM. Western blot revealed that vesicles were positive for exosomal markers (Alix, TSG101, CD63), but did not express cytoplasmic biomarker GM130 (Fig. 1B). NTA analysis verified that the diameter of vesicles was approximately 100 nm (Fig. 1C). Meanwhile, the exosomes were seen as double-concave disks when observed by TEM (Fig. 1D). Next, the PKH67-labeled exosomes were incubated with ECs; the presence of green fluorescence distributed around the nuclei indicated that M0-exos and M2-exos could be internalized by ECs (Fig. 1E–F).

**Fig. 2:**
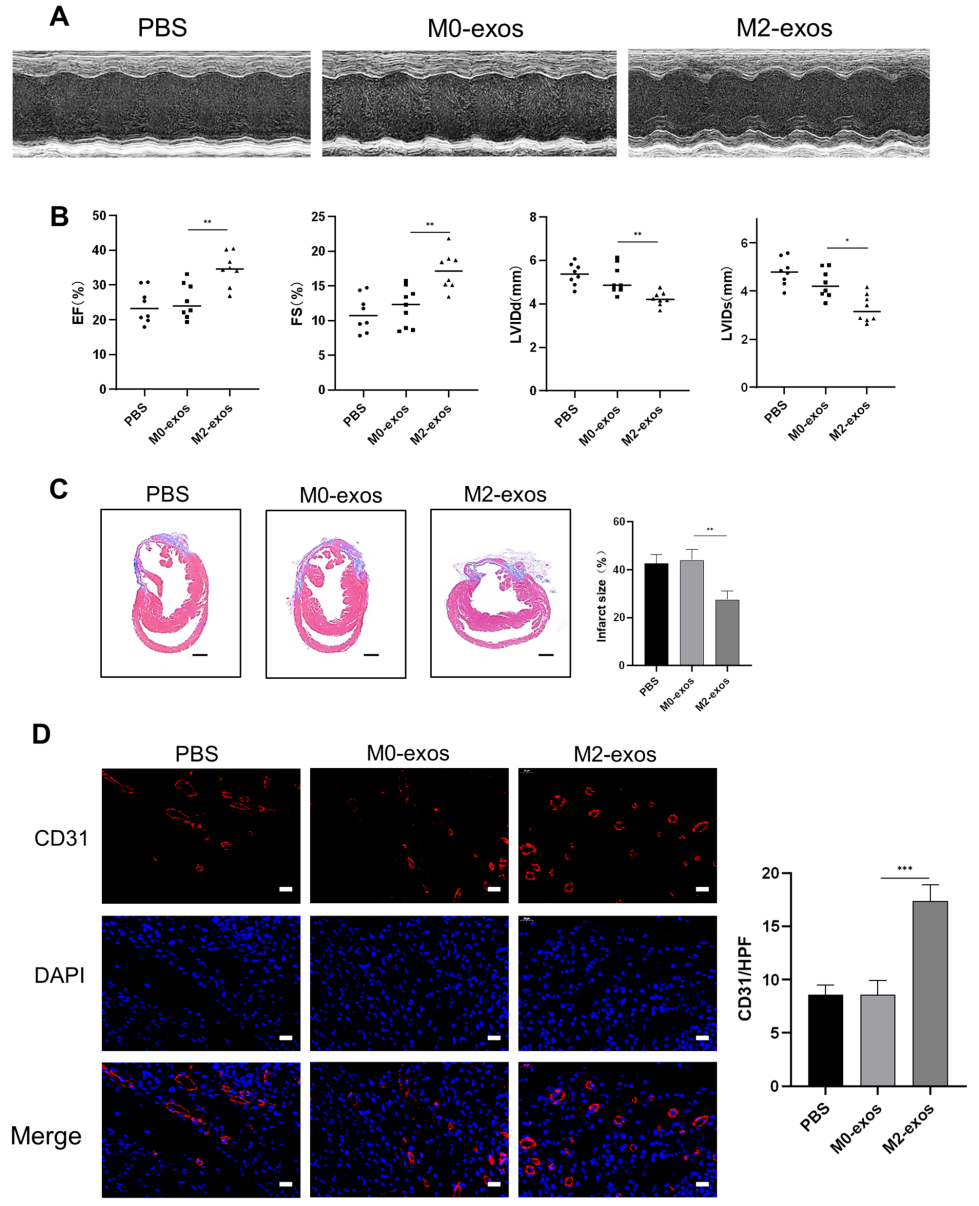
M2-exos improved cardiac function and enhanced angiogenesis after MI. Mice were treated with M0-exos, M2-exosomes, or PBS through intramyocardial injections after MI. (**A**) Representative echocardiographic images of mice 4 weeks after MI injury and treatment. (**B**) Echocardiographic assessments of LVEF, FS, LVIDd, and LVIDs, *n* = 8. (**C**) Infarct size was measured using Masson trichrome staining, scale bar = 1 mm, *n* ≥ 4. (**D**) Representative images of CD31 immunofluorescence staining in the border zone of infarcted heart, scale bar = 20 μm, *n* ≥ 4. (Data are presented as mean ± SD; *P < 0.05, **P < 0.01, ***P < 0.001.)

To assess the effect of M2-exos on angiogenesis after MI, M0-exos or M2-exos were delivered through intramyocardial injection at the onset of MI. The ST-segment elevation in ECG and the consistency of infarct size among groups confirmed the successful establishment of MI model (Supplementary material online, Figure S2A–B). Four weeks following MI, the LVIDd and LVIDs were significantly decreased in the M2-exos groups; the LVEF and FS markedly increased along with ventricular systolic function (Fig. 2A–B). However, the M0-exos had no clear effects on these parameters relative to PBS groups. Furthermore, M2-exos decreased the proportion of infarct size as observed by Masson trichrome staining (Fig. 2C). Most importantly, angiogenesis increased following M2-exos treatment as shown by higher numbers of CD31^+^ cells in the border zone (Fig. 2D). Taken together, M2-exos treatment after MI improved the recovery of cardiac function.

### M2-Exos accelerated angiogenesis In Vitro

**Fig. 3:**
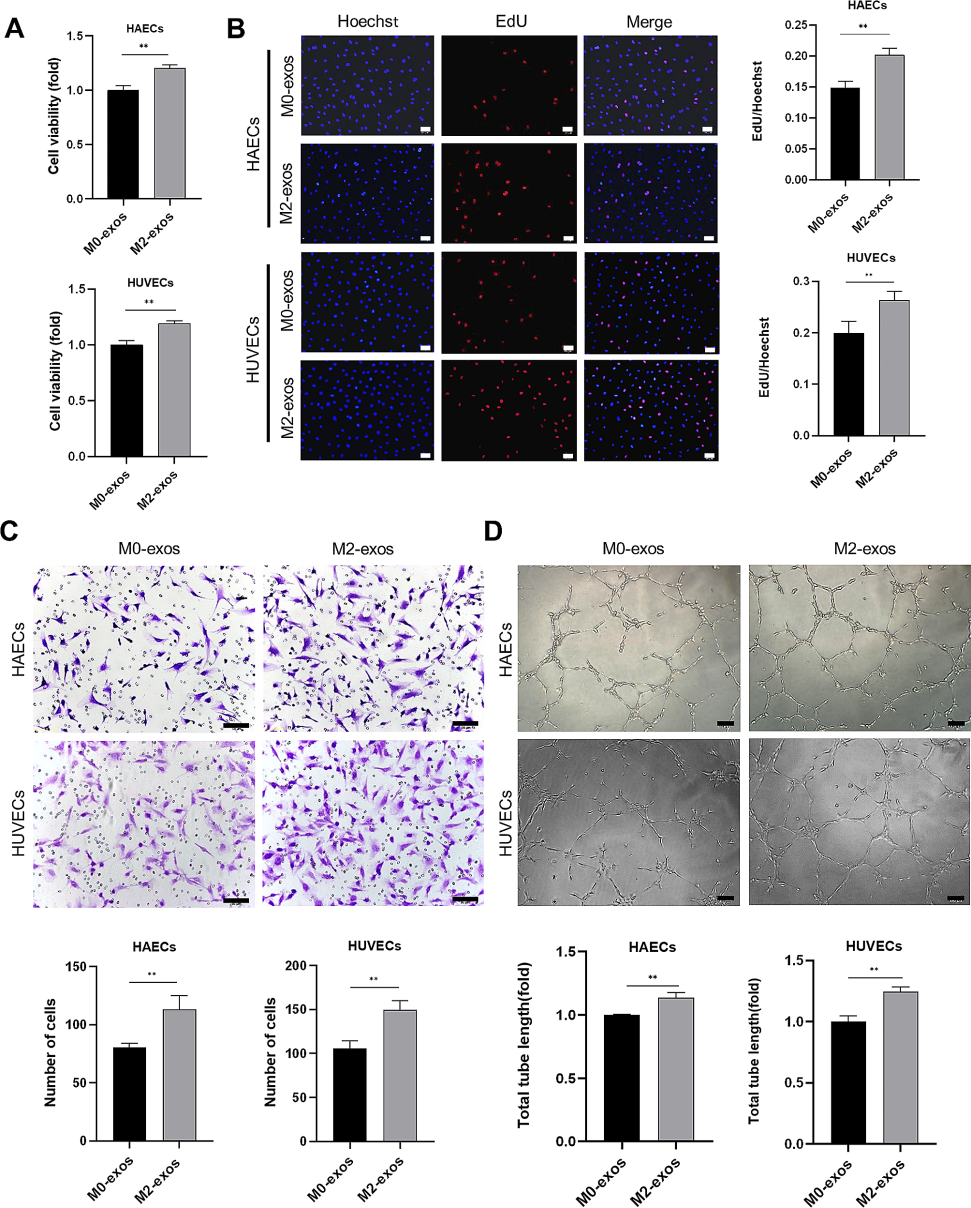
M2-exos promoted angiogenesis in vitro. HAECs and HUVECs were treated with exosomes for 48 h. (**A**) Cell viability detection of HAECs and HUVECs under exosomes treatment using MTS assay. (**B**) Proliferation detection of HAECs and HUVECs using EdU assay, scale bar = 50 μm. (**C**) Migration detection of HAECs and HUVECs using Transwell assay, scale bar = 50 μm. (**D**) Tube formation assay of HAECs and HUVECs, scale bar = 100 μm. (Data are presented as mean ± SD; *n* = 3; **P < 0.01.)

Since biomolecules may have diverse roles in distinct ECs, we used primary HAECs and HUVECs to confirm the direct effect and mechanism of action of M2-exos. The angiogenic ability of ECs was examined after treatment with M0-exos and M2-exos, including cell viability, proliferation, migration, and tube formation. Cell viability was determined using MTS assay, which revealed that M2-exos improved EC viability compared to M0-exos (Fig. 3A). The EdU assay showed that the proliferation rate of ECs increased significantly after treatment with M2-exos (Fig. 3B). Then, the motility of ECs was tested by Transwell assay and we found that M2-exos promoted the migration of HAECs and HUVECs (Fig. 3C). We subsequently evaluated whether M2-exos regulate EC angiogenic ability using Matrigel. The results presented in Fig. 3D indicate that ECs stimulated with M2-exos induced greater tube formation. These results provide evidence that M2-exos promote the cardinal features of angiogenesis after MI, including EC viability, proliferation, migration, and tube formation.

### The molecules loaded in the vesicles were responsible for the Proangiogenic effects of M2-Exos

**Fig. 4:**
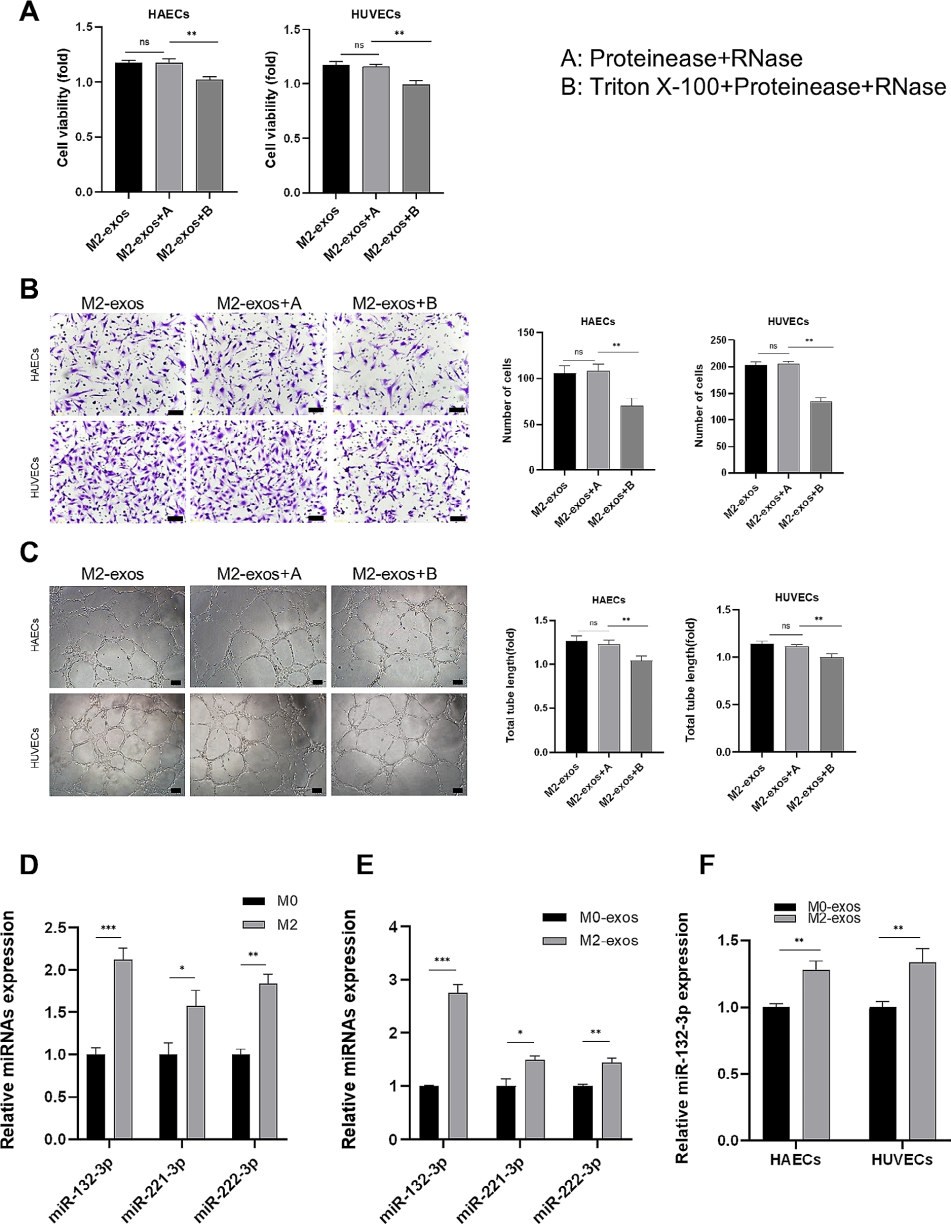
The molecules loaded in the vesicles were responsible for the proangiogenic effects of M2-exos. HAECs and HUVECs were treated with processed exosomes for 48 h. (**A**) Cell viability detection of HAECs and HUVECs using MTS assay. (**B**) Migration detection of HAECs and HUVECs using Transwell assay, scale bar = 50 μm. (**C**) Tube formation assay of HAECs and HUVECs, scale bar = 100 μm. (**D**) Relative miRNA expression in macrophages was detected by RT-PCR. (**E**) Relative miRNA expression in macrophage-derived exosomes was detected by RT-PCR. (**F**) Relative miR-132-3p expression in HAECs and HUVECs was detected by RT-PCR. A: Proteinease + RNase, B: Triton X-100 + Proteinease + RNase. (Data are presented as mean ± SD; *n* = 3; *P < 0.05, **P < 0.01, ***P < 0.001.)

In an attempt to investigate the underlying mechanism of M2-exos-induced angiogenesis, we treated M2-exos with RNase and protease to digest the RNAs/proteins that were free-floating and located on the surface of M2-exos, while Triton X-100 was used simultaneously to eliminate all RNA and protein. As shown in Fig. 4A–C, M2-exos after RNase and protease treatment presented no clear change in the angiogenic capability of ECs compared with M2-exos. However, the synchronous treatment of Triton X-100 decreased the proangiogenic effect of M2-exos. These findings indicate that the proangiogenic effects of M2-exos depended on its internal molecules.

To unveil the potential molecular mechanisms of the proangiogenic role of M2-exos, we analyzed the profile of miRNA published in GEO datasets. GSE97467, a microarray analysis of M0 and M2 macrophage-derived exosomes was selected and analyzed using GEO2R (Supplementary material online, Table S2). It was found that three angiogenic miRNAs were elevated in M2-exos, including miR-132-3p, miR-221-3p, and miR-222-3p. Among them, miR-132-3p was the most abundant miRNA in M2 macrophages as well as their exosomes, which was validated by RT-qPCR (Fig. 4D–E). As M1 macrophage may exert contrary functions to M2, we also confirmed that miR-132-3p was not abundant in M1 macrophage and its exosomes (Supplementary material online, Figure S3A–B). Furthermore, we found that the relative expression level of miR-132-3p was increased in ECs after treatment with M2-exos, suggesting that M2-exos transfer miR-132-3p into ECs (Fig. 4F). These data demonstrate that M2-exos-induced angiogenesis that was mediated at least in part by miRNAs.

### M2-Exos-Induced Angiogenesis is mediated in part by MiR-132-3p

We transfected miR-132-3p mimics or inhibitors to clarify the function of miR-132-3p in ECs. After determining the transfection efficiency in HAECs and HUVECs (Supplementary material online, Figure S4), we observed that the cell viability, proliferation, migration, and tube formation of ECs were enhanced by miR-132-3p mimics, and attenuated by inhibitors (Supplementary material online, Figure S5). Therefore, miR-132-3p improved the angiogenic ability of ECs.

**Fig. 5:**
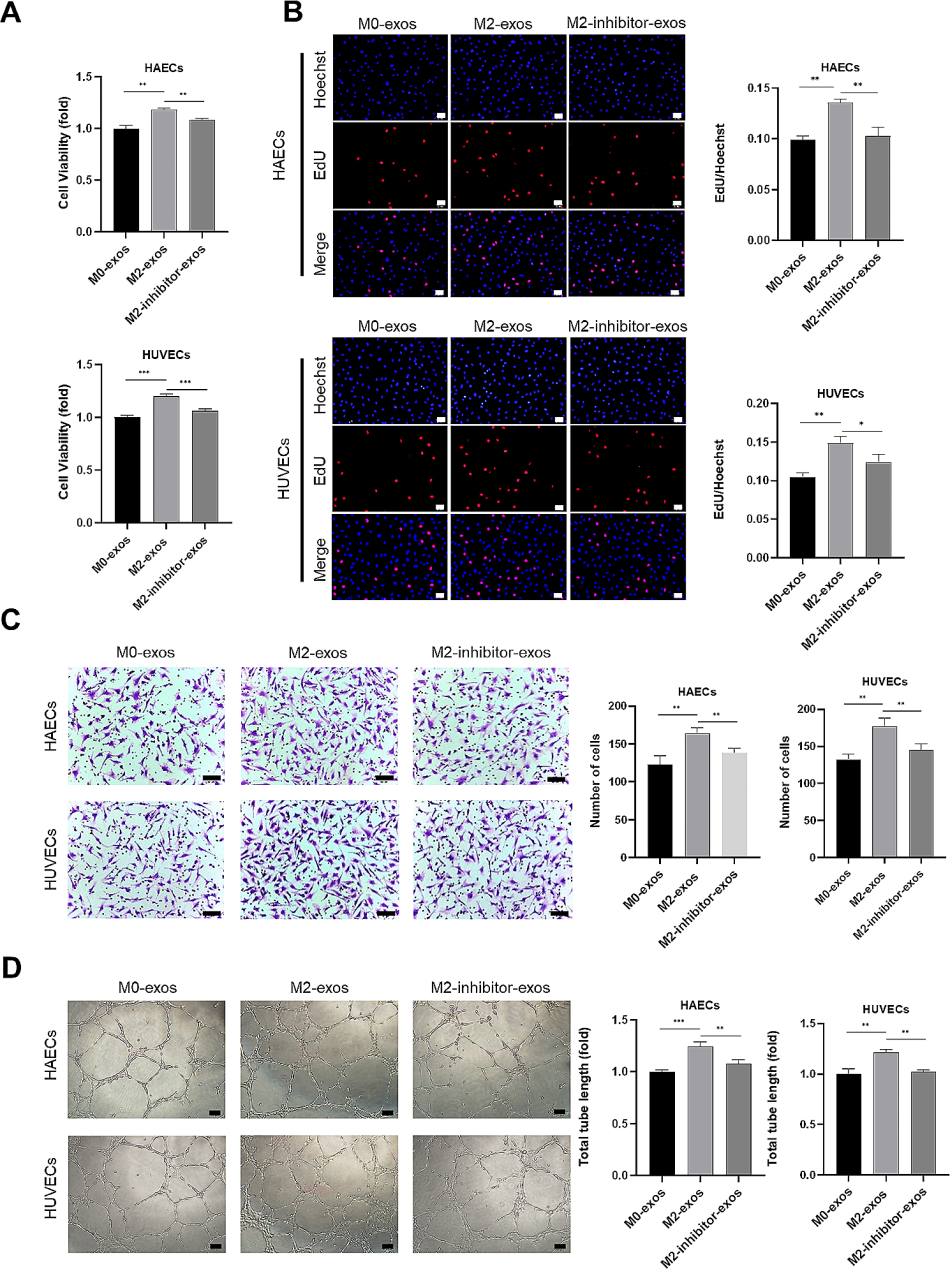
Inhibition of miR-132-3p attenuates the beneficial effects of M2-exos on angiogenesis. HAECs and HUVECs were treated with M0-exos, M2-exos, or M2-inhibitor-exos for 48 h. (**A**) MTS assay was used to assess the viability of HAECs and HUVECs treated with M2-exos with reduced miR-132-3p level. (**B**) EdU assay was used to evaluate the proliferation of HAECs and HUVECs treated with exosomes, scale bar = 50 μm. (**C**) Transwell assay was used to evaluate the migration of HAECs and HUVECs, scale bar = 50 μm. (**D**) Tube formation assay of HAECs and HUVECs, scale bar = 100 μm. (Data are presented as mean ± SD; *n* = 3; *P < 0.05, **P < 0.01, ***P < 0.001.)

To identify the effect of miR-132-3p within the angiogenic process of M2-exos, we next transfected M2 macrophages with miR-132-3p inhibitor and isolated their exosomes (M2-inhibitor-exos). RT-qPCR verified the markedly decreased miR-132-3p levels after transfection (Supplementary material online, Figure S6). After incubation with ECs, the inhibitor of miR-132-3p significantly reduced the enhanced angiogenic effects of M2-exos (Fig. 5A–D). Together, these results suggested that miR-132-3p-enriched exosomes promoted the angiogenesis of ECs.

### M2-Exos containing MiR-132-3p promote angiogenesis following MI

**Fig. 6:**
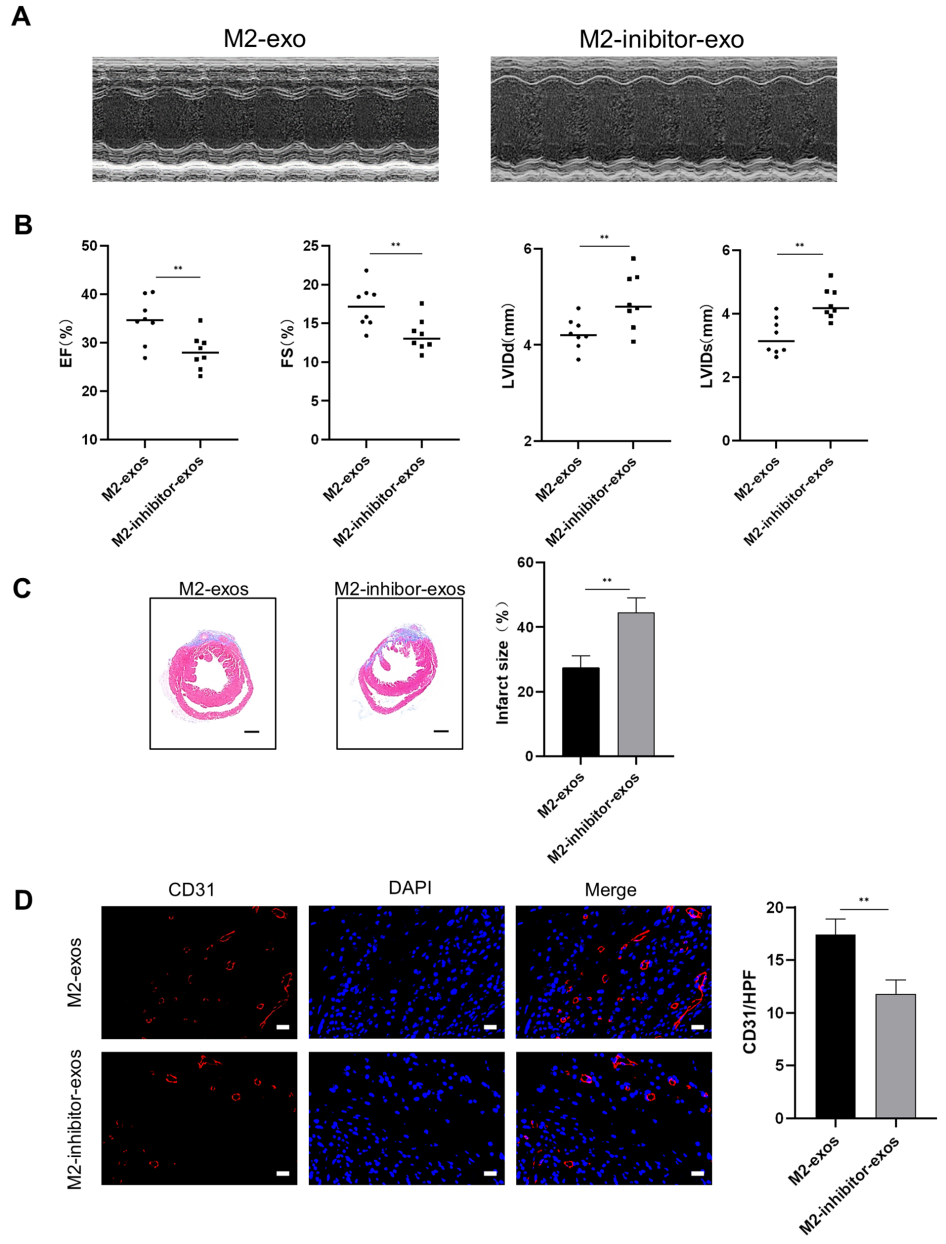
Inhibition of miR-132-3p attenuates the effects of M2-exos on cardiac repair. Mice were treated with M2-exos or M2-exos with reduced miR-132-3p level through intramyocardial injections after MI. (**A**) Representative echocardiographic images of cardiac function of mice 4 weeks after MI. (**B**) Echocardiographic assessments of LVEF, FS, LVIDd, and LVIDs, *n* = 8. (**C**) Masson trichrome staining was used to quantify the infarct size, scale bar = 1 mm, *n* ≥ 4. (**D**) Immunofluorescence staining of CD31 in the border zone of infarcted heart, scale bar = 20 μm, *n* ≥ 4. (Data are presented as mean ± SD; **P < 0.01.)

To further investigate the effect of miR-132-3p on the M2-exos-induced angiogenesis after MI, the M2-exos or M2-inhibitor-exos was delivered through intramyocardial injection at the onset of MI. As shown in Fig. 6 (A–C), treatment with M2-inhibitor-exos markedly decreased the LVEF and FS, but increased the LVIDd and LVIDs, and expanded infarct size after four weeks compared with M2-exos. Furthermore, the CD31^+^ cells in the border zone were significantly decreased in the M2-inhibitor-exos group (Fig. 6D). Collectively, miR-132-3p is required for the cardioprotective effects of M2-exos.

### MiR-132-3p regulated *THBS1* expression by targeting its 3´UTR

**Fig. 7:**
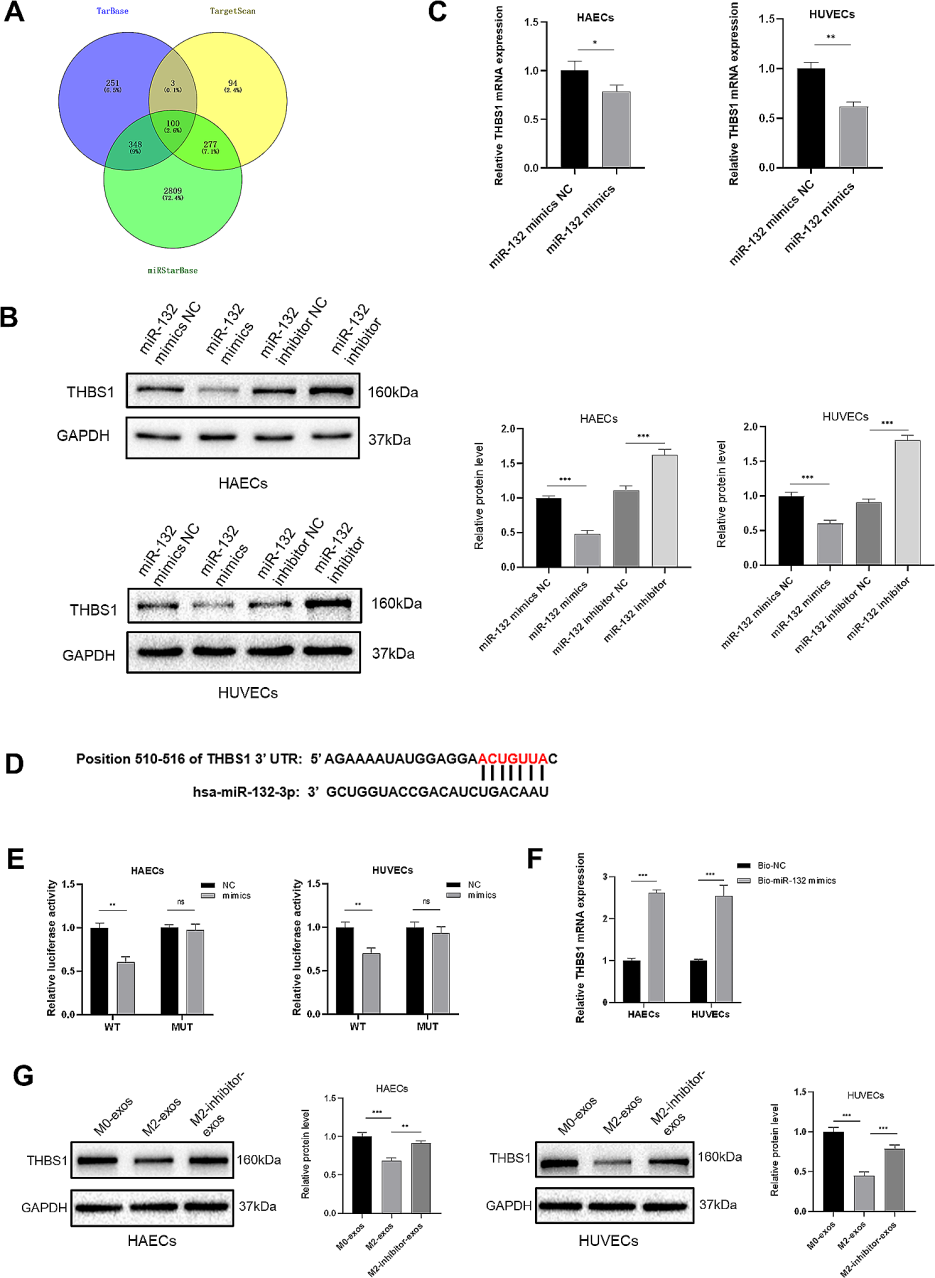
THBS1 is a direct target of miR-132-3p. (**A**) The intersection of the target genes of miR-132-3p among three datasets. (**B**) Western blot analysis was used to assess the expression of potential target genes of *THBS1* after transfecting ECs with miR-132-3p mimics or inhibitors. (**C**) The mRNA level of *THBS1* was determined in HAECs and HUVECs transfected with miR-132-3p mimics. (**D**) The predicted binding sites of miR-132-3p and *THBS1* 3´UTR through TargetScan. (**E**) Dual-luciferase report assays were conducted to confirm the binding site of miR-132-3p and *THBS1* 3´UTR. (**F**) miRNA pull-down assay was performed to validate the combination of miR-132-3p and *THBS1*. (**G**) Western blot analysis was used to confirm that M2-exos regulated *THBS1* expression partially through miR-132-3p. (WT: wild-type, MUT: mutant-type. Data are presented as mean ± SD; *n* = 3; *P < 0.05, **P < 0.01, ***P < 0.001.)

Next, three online bioinformatics tools (TarBase, TargetScan, and miRStarBase) were used to predict the possible targets of miR-132-3p, and 100 genes were identified in the intersection of three databases (Fig. 7A). After reviewing the functional reports of these candidate genes, we noted that *THBS1*, *SPRED1*, *ARID1A*, *PTEN* might be the targets of miR-132-3p. To determine the regulatory effect of miR-132-3p on these candidates, miR-132-3p mimics or inhibitors were transfected into ECs. The results presented in Fig. 7B and Figure S7 indicate that only *THBS1* could be negatively regulated by miR-132-3p at the protein level. Additionally, the mRNA levels of *THBS1* were significantly reduced by miR-132-3p mimics (Fig. 7C). We subsequently performed the dual-luciferase report assays to confirm the relationship between the *THBS1* and miR-132-3p. The 3**´**UTR region of *THBS1* mRNA that contained the predicted binding sites for miR-132-3p was cloned and inserted into a luciferase reporter plasmid, and another mutant *THBS1* 3**´**UTR-driven vector was conducted (Fig. 7D). As shown in Fig. 7E, co-transfection of wild-type luciferase vector with miR-132-3p mimics significantly decreased the luciferase activity compared to the control group. However, the suppression was abrogated when the predicted binding sites for miR-132-3p were mutated. Moreover, the miRNA pull-down assay was performed to further validate that *THBS1* is a direct target of miR-132-3p. The *THBS1* mRNA pulled-down by biotin-labeled mimics was markedly higher than that in the control group, indicating that miR-132-3p could directly bind to *THBS1* (Fig. 7F). We assessed the regulatory effect of M2-exos on *THBS1* and found incubation with M2-exos significantly reduced the protein level of *THBS1* of ECs compared with M0-exos, while the inhibitory effect was reversed with reduced miR-132-3p level in M2-exos (Fig. 7G). Altogether, these data show that M2-exos regulate the expression of *THBS1* through the direct binding of miR-132-3p to its 3**´**UTR.

### MiR-132-3p promoted angiogenesis by targeting *THBS1*

**Fig. 8:**
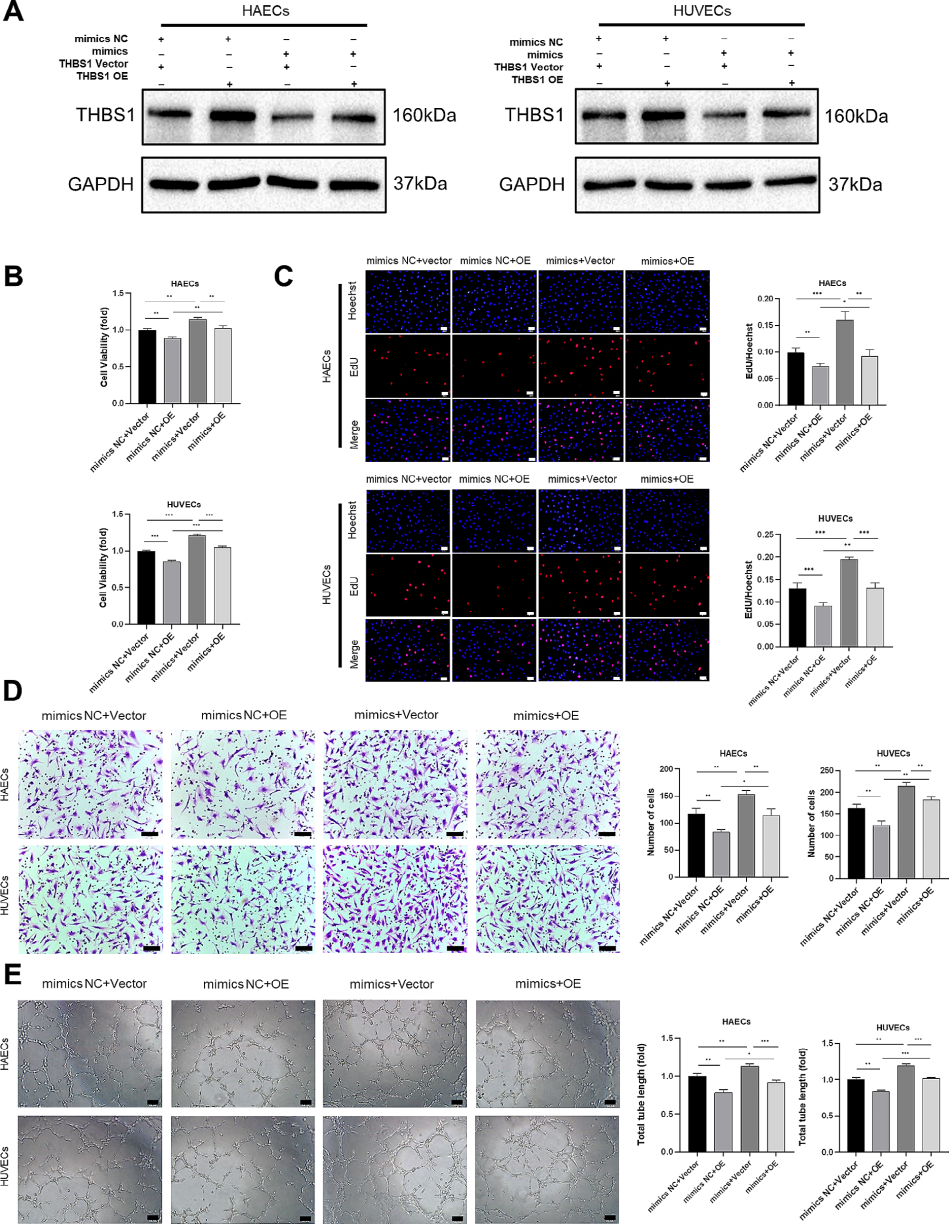
MiR-132-3p promoted angiogenesis by targeting *THBS1*. HAECs and HUVECs were co-transfected with miR-132-3p mimics and *THBS1* overexpression plasmid for 48 h. (**A**) Western blot analysis was used to assess the expression of *THBS1* in HAECs and HUVECs after co-transfection. (**B**) MTS assay was used to detect the viability of transfected HAECs and HUVECs. (**C**) EdU assay was used to determine the proliferation of transfected HAECs and HUVECs, scale bar = 50 μm. (**D**) Transwell assay was used to evaluate the migration of transfected HAECs and HUVECs, scale bar = 50 μm. (**E**) Tube formation assay of transfected HAECs and HUVECs, scale bar = 100 μm. (Data are presented as mean ± SD; *n* = 3; *P < 0.05, **P < 0.01, ***P < 0.001.)

Subsequently, we assessed whether *THBS1* is involved in the angiogenic effect of miR-132-3p. To confirm this, western blotting was used to evaluate the level of THBS1 protein; we found that the successful up-regulation of *THBS1* in ECs, and overexpression of *THBS1* could reverse the suppression by miR-132-3p mimics (Fig. 8A). Consistent with the results of western blots, as shown in Fig. 8B–E, the overexpression of *THBS1* significantly attenuated the cell viability, proliferation, migration, and tube formation of ECs, and the angiogenic effects of miR-132-3p mimics were largely reversed by *THBS1* overexpression. Therefore, our results further established that miR-132-3p promoted angiogenesis by targeting *THBS1*.

## Discussion

It is well established that macrophages play a vital role in angiogenesis after MI and exosomes tightly regulate and balance the crosstalk between different cells for cardiac repair [16–18]. Here, we hypothesized that M2-exos exhibited a cardioprotective effect in myocardial injury and clearly demonstrated that M2-exos promote cardinal features of angiogenesis after MI, including EC viability, proliferation, migration, and tube formation. Kim et al. isolated exosomes from M2 macrophages and found that M2-exos induced a conversion of M1 macrophages to M2 macrophages and accelerated wound healing by improving angiogenesis [19]. However, we confirmed the direct effect of M2-exos on two distinct ECs and validated that the molecules loaded in the vesicles were responsible for the angiogenic effects of M2-exos. Furthermore, the higher abundance of miR-132-3p in M2-exos was required for their cardioprotective effects. Mechanistically, M2-exos regulate the expression of *THBS1* through the direct binding of miR-132-3p to its 3´UTR, which regulated the angiogenic ability of ECs. The present study provides novel insights into the effects of M2 macrophages in MI and suggests the application of M2-exos as a potential novel therapeutic strategy in myocardial ischemia.

Therapeutic angiogenesis is associated with better outcomes in MI, as evidenced by improved cardiac function, smaller infarcted areas, and less remodeling, indicating that endogenous angiogenic response is somehow limited and can be intensified to attain a therapeutic effect [20, 21]. Recently, studies have focused on the involvement of exosomes in angiogenesis after MI. Exosomes derived from stem cells are a potential treatment strategy in MI because of their advantages of easy storage and the fact that they can be directly delivered into ischemic heart tissue [17, 22]. Moreover, the intercellular communication in MI microenvironment mediated by exosomes plays a critical role in angiogenesis and cardiac repair [13]. Although macrophages have emerged as main driving factors of the angiogenic response after MI, it was previously shown that M1 macrophage-derived exosomes (M1-exos) suppress angiogenesis and exacerbate cardiac dysfunction after myocardial ischemia [23]. Our study demonstrated opposite effects of M2-exos versus M1-exos; this suggests that the biological activities of exosomes are similar to those of the donor cells, and the balance between different macrophage subsets is necessary for cardiac repair.

Although different methods for exosome extraction have been developed, the obtained exosomes were inevitably contaminated with RNAs/proteins that were free-floating and located on the surface of exosomes [24, 25]. Therefore, these dopants should be taken into consideration when evaluating the biological functions of extracted exosomes. However, studies on the biological impact of exosomes always sidestepped this issue. For this reason, we treated M2-exos with RNase and protease to digest the potential dopants according to the latest guidelines in extracellular vesicle research [25] and demonstrated that the molecules loaded in the vesicles were responsible for the angiogenic effects of M2-exos.

Although we provide evidence that M2-exos improved cardiac function and validated the direct biological effects of M2-exos on ECs, which constitutes an important contribution to the elucidation of the role of M2 macrophages in intercellular communication in post-infarction angiogenesis, we did not explore the impact of M2-exos on cardiomyocytes. Previous research has demonstrated that M2-exos promote viability of hypoxia/reoxygenation-treated cardiomyocytes and alleviate myocardial ischemia/reperfusion injury in vivo [26]. Similarly, studies carried out by Long et al. illustrated that M2-exos suppressed apoptosis and improved viability of hypoxia-induced cardiomyocytes and promoted cardiac repair in myocardial ischemia [27]. Therefore, studying the effects and mechanism of action of M2-exos in intercellular communication in cardiovascular diseases is expected to open new research avenues in cardiac repair after MI and injury.

Previous studies have shown that changes in the miRNA profile of exosomes under physiological and pathological conditions was finely orchestrated to modulate the bioactivity of target cells [28]. It is conceivable that exosomal miRNAs exert a critical role in cardiovascular diseases by directly binding to target genes [29]. miR-132-3p is a typical highly conserved and multifunctional miRNA, transcribed from an intergenic region on human chromosome 17, known for its bioactivity in multiple diseases, such as myocardial ischemia [30, 31]. It was previously shown that miR-132 level was increased in Ang II-treated cardiac fibroblasts and the MI-induced heart failure, and this improved cardiac function and enhanced cardiac fibrosis by inhibiting PI3K/Akt pathway [32]. According to our model, the higher abundance of miR-132-3p in M2-exos exerts an angiogenic effect and recapitulates the function of M2-exos in cardiac repair. Importantly, the effects of miR-132-3p in vitro were validated in two distinct ECs with similar functions. Studies carried out by Ribeiro-Rodrigues et al. found that miR-222 and miR-143 were enriched in the exosomes secreted by H9c2 cells under ischemia-mimetic conditions [13]. However, the effect of each of the two miRNAs was apparently different in distinct ECs, suggesting that miRNAs may play diverse roles in different vessels. Therefore, the functions of biomolecules need to be carefully screened in different ECs to avoid misinterpretation of data when using special basic conditions.

In addition to miR-132-3p, it is likely that the angiogenic effects of M2-exos on ECs depends on other miRNAs enriched in M2-exos. Studies indicate that miR-222 was relatively the most abundant miRNA in exosomes, which exerts angiogenic effects after MI [13]. Furthermore, other biomolecules, such as long noncoding RNAs or proteins could also serve as key effectors driving the angiogenic abilities of M2-exos, which should be investigated in future studies.

In the present study, we identified *THBS1* as a direct target of miR-132-3p through miRNA gene BLAST in three independent online bioinformatics tools, and verified this by dual-luciferase reporter and RNA pull-down assays. Overexpression of miR-132-3p was found to suppress *THBS1* mRNA and protein levels through binding to the 3´UTRof *THBS1*. Similarly, M2-exos down-regulate the expression of *THBS1*; this inhibitory effect was reversed by M2-inhibitor-exos treatment. Moreover, reintroduction of *THBS1* in the presence of miR-132-3p mimics rescued the angiogenic effect of miR-132-3p on ECs. Therefore, we illustrated a new regulatory mechanism of action for miR-132-3p and established that the augmentation effects of M2-exos might be achieved by targeting *THBS1*. However, we did not investigate the impact of *THBS1* on angiogenesis after MI. A mutually antagonistic link between *THBS1* and nitric oxide signaling has previously been demonstrated, suggesting that targeting *THBS1* may prove beneficial [32]. Therefore, the role of *THBS1* in angiogenesis after MI needs to be further clarified using conditional knockout mice.

## Conclusion

In conclusion, to the best of our knowledge, this is the first study to propose that M2-exos deliver miR-132-3p to ECs, and enhanced the angiogenic ability of ECs by down-regulating *THBS1*, thereby promoting angiogenesis after myocardial infarction. This may serve as a potential novel therapeutic approach for cardiac repair.

### Electronic supplementary material

Below is the link to the electronic supplementary material.

**Fig S1.** The effect of M2 macrophages on angiogenesis was attenuated in the absence of exosomes. scale bar = 100 μm; **P < 0.01
**Fig S2.** Establishment of MI model. (A) ST-segment elevation in ECG after left anterior descending ligation. (B) Consistency of infarct size among groups
**Fig S3.** Relative miR-132-3p expression in M1 macrophages (A) and their derived exosomes (B)
**Fig S4.** MiR-132-3p level was detected in the transfected HAECs (A) and HUVECs (B); ***P < 0.001, ****P < 0.0001
**Fig S5.** Mir-132-3p promoted the angiogenic ability of ECs. HAECs and HUVECs were transfected with miR-132-3p mimics or inhibitors for 48 h (A) MTS assay was used to evaluate the viability of transfected HAECs and HUVECs. (B) EdU assay was used to measure the proliferation of transfected HAECs and HUVECs, scale bar = 50 μm. (C) Transwell assay was used to evaluate the migration of transfected HAECs and HUVECs, scale bar = 50 μm. (D) Tube formation assay of transfected HAECs and HUVECs, scale bar = 100 μm. (Data are presented as mean ± SD; *n* = 3; *P < 0.05, **P < 0.01, ***P < 0.001
**Fig S6.** MiR-132-3p level was detected in M2 macrophages (A) and their derived exosomes (B) after transfection with miR-132-3p inhibitor; ***P < 0.001
**Figure S7.** Western blot analysis was used to assess the expression of potential target genes after transfecting ECs with miR-132-3p mimics or inhibitors.

## Data Availability

All the raw data of this work are available from the corresponding author (Rongchong Huang) upon reasonable request.

## Abbreviations

MI: myocardial infarction
M2-exos: M2 macrophage-derived exosomes
ECs: endothelial cells
miRNAs: microRNAs
HAECs: human artery endothelial cells
HCAECs: human coronary artery endothelial cells
HUVECs: human umbilical vein endothelial cells
ECM: endothelial cell medium
FBS: fetal bovine serum
NTA: nanoparticle tracking analysis
TEM: transmission electron microscopy
LVEF: left ventricular ejection fraction
FS: fractional shortening
TTC: triphenyltetrazolium chloride
CM: conditioned medium
