# Supplementary material for: M2 macrophage-derived exosomes promote angiogenesis and improve cardiac function after myocardial infarction
Source: Biol Direct. 2024 Jun 6;19:43. doi: 10.1186/s13062-024-00485-y (PMC11155164; doi:10.1186/s13062-024-00485-y)
Supplement: Supplementary file 1 — Fig S1. The effect of M2 macrophages on angiogenesis was attenuated in the absence of exosomes. scale bar = 100 μm; **P < 0.01 Fig S2. Establishment of MI model. (A) ST-segment elevation in ECG after left anterior descending ligation. (B) Consistency of infarct size among groups Fig S3. Relative miR-132-3p expression in M1 macrophages (A) and their derived exosomes (B) Fig S4. MiR-132-3p level was detected in the transfected HAECs (A) and HUVECs (B); ***P < 0.001, ****P < 0.0001 Fig S5. Mir-132-3p promoted the angiogenic ability of ECs. HAECs and HUVECs were transfected with miR-132-3p mimics or inhibitors for 48 h (A) MTS assay was used to evaluate the viability of transfected HAECs and HUVECs. (B) EdU assay was used to measure the proliferation of transfected HAECs and HUVECs, scale bar = 50 μm. (C) Transwell assay was used to evaluate the migration of transfected HAECs and HUVECs, scale bar = 50 μm. (D) Tube formation assay of transfected HAECs and HUVECs, scale bar = 100 μm. (Data are presented as mean ± SD; n = 3; *P < 0.05, **P < 0.01, ***P < 0.001 Fig S6. MiR-132-3p level was detected in M2 macrophages (A) and their derived exosomes (B) after transfection with miR-132-3p inhibitor; ***P < 0.001 Figure S7. Western blot analysis was used to assess the expression of potential target genes after transfecting ECs with miR-132-3p mimics or inhibitors. [file 13062_2024_485_MOESM1_ESM.docx]

**SUPPLEMENTARY DATA**

**M2 macrophages-derived exosomes promote angiogenesis and improve cardiac function after myocardial infarction**

Table S1. Primer oligonucleotide sequences.

| Gene | Primers |
| --- | --- |
| Arg1 | F: TGGACAGACTAGGAATTGGCA |
|  | R: CCAGTCCGTCAACATCAAAACT |
| CD206 | F: GGGTTGCTATCACTCTCTATGC |
|  | R: TTTCTTGTCTGTTGCCGTAGTT |
| IL-10 | F: GACTTTAAGGGTTACCTGGGTTG |
|  | R: TCACATGCGCCTTGATGTCTG |
| THBS1  hsa-miR-132-3p  hsa-miR-221-3p  hsa-miR-222-3p  U6  GAPDH | AGACTCCGCATCGCAAAGG  TCACCACGTTGTTGTCAAGGG  F: ACACTCCAGCTGGGTAACAGTCTACAGCCA  R: TGGTGTCGTGGAGTCG  F: ACACTCCAGCTGGGAGCTACATTGTCTGCTG  R: TGGTGTCGTGGAGTCG  F: ACACTCCAGCTGGGAGCTACATCTGGCTA  R: TGGTGTCGTGGAGTCG  F: AAAGCAAATCATCGGACGACC  R: GTACAACACATTGTTTCCTCGGA  F: GGAGCGAGATCCCTCCAAAAT  R: GGCTGTTGTCATACTTCTCATGG |

| miRNA | *p* Value | Log (FC) |
| --- | --- | --- |
| miR-4539 | 0.000214 | 4.6507 |
| miR-548f | 0.000237 | 5.51911 |
| miR-708-5p | 0.000652 | 6.25732 |
| miR-1911-5p | 0.001523 | 6.70128 |
| miR-135b-3p | 0.003422 | 5.4381 |
| miR-503-5p | 0.007761 | 6.06577 |
| miR-939-3p | 0.00839 | 4.22962 |
| miR-132-3p | 0.022509 | 6.15258 |
| miR-542-5p | 0.022509 | 5.45268 |
| miR-222-3p | 0.027651 | 4.77063 |
| miR-302c-5p | 0.03109 | 3.69201 |
| miR-186-5p | 0.03109 | 3.71041 |
| miR-221-3p | 0.03873 | 6.71738 |

Table S2. Top 13 upregulated miRNAs in GSE97467


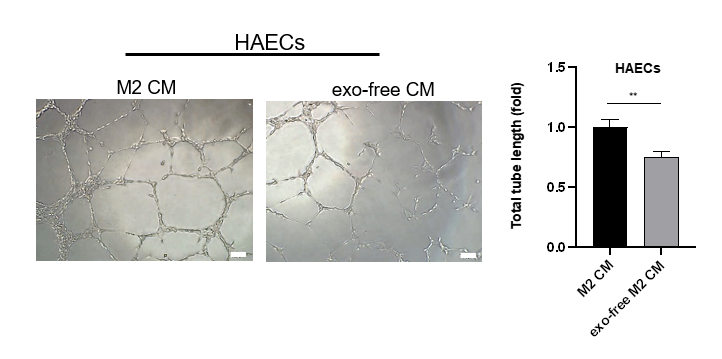


**Figure S1.** The effect of M2 macrophages on angiogenesis was attenuated in the absence of exosomes, scale bar=100μm, ***P*<0.01.


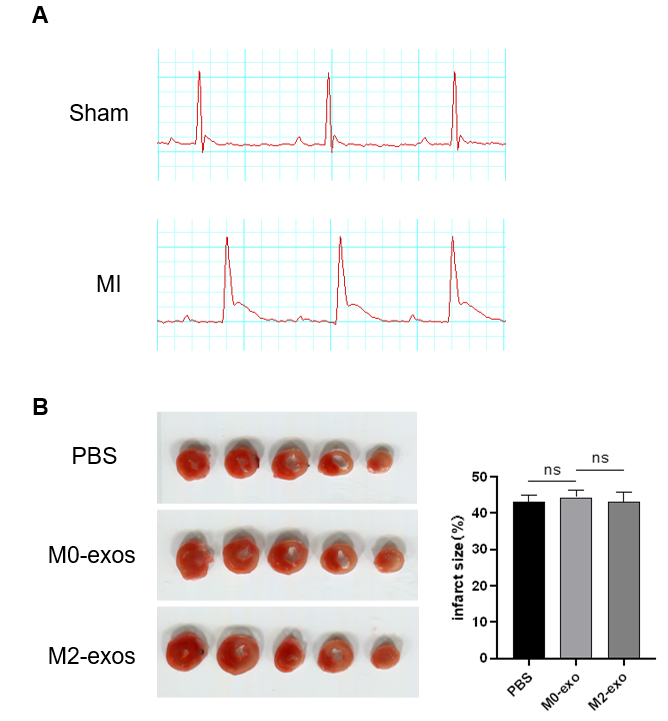


**Figure S2.** Establishment of MI model. (A) ST-segment elevation in ECG after left anterior descending ligation. (B) Consistency of infarct size among groups.

**
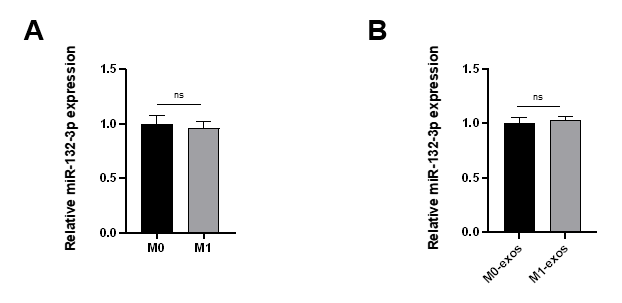
****Figure S3.** Relative miR-132-3p expression in M1 macrophages (A) and their derived exosomes (B).


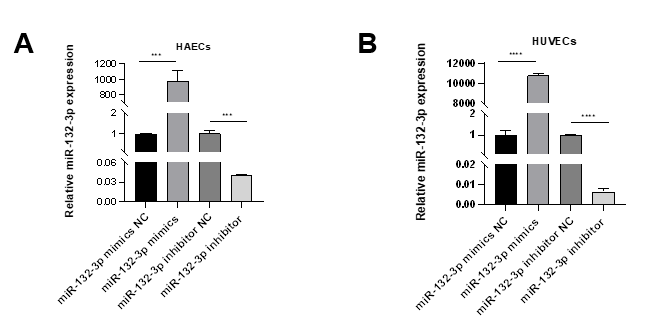


**Figure S4.** miR-132-3p level was detected in the transfected HAECs (A) and HUVECs (B), ****P*<0.001, *****P*<0.0001.


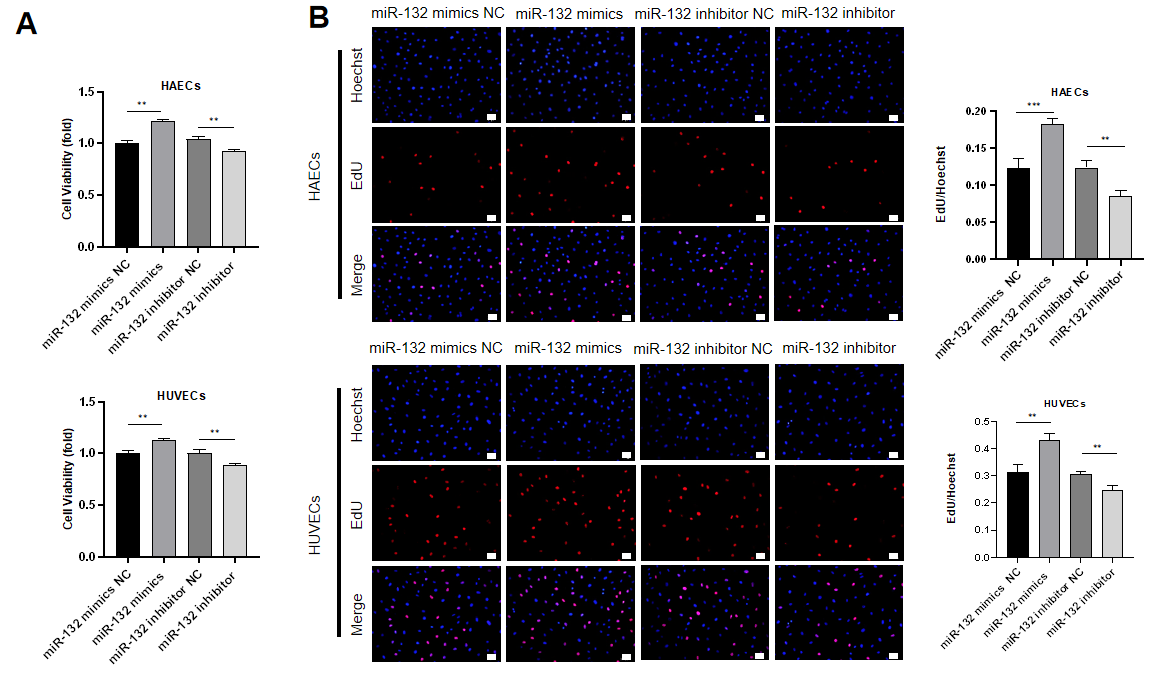


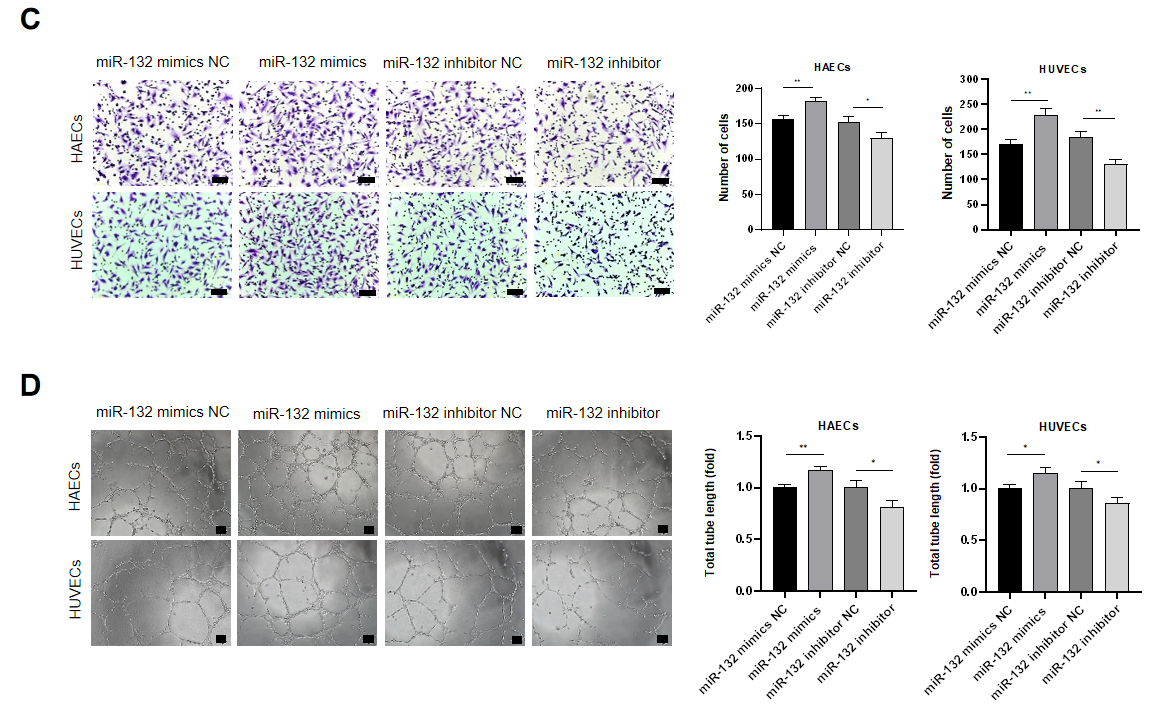


**Figure S5.** Mir-132-3p promoted the angiogenic ability of ECs. HAECs and HUVECs were transfected with miR-132-3p mimics or inhibitors for 48 h (A) MTS assay was used to evaluate the viability of transfected HAECs and HUVECs. (B) EdU assay was used to measure the proliferation of transfected HAECs and HUVECs, scale bar=50 μm. (C) Transwell assay was used to evaluate the migration of transfected HAECs and HUVECs, scale bar=50 μm. (D) Tube formation assay of transfected HAECs and HUVECs, scale bar=100 μm. (Data are presented as mean±SD; n=3; *P<0.05, **P<0.01, ***P<0.001.


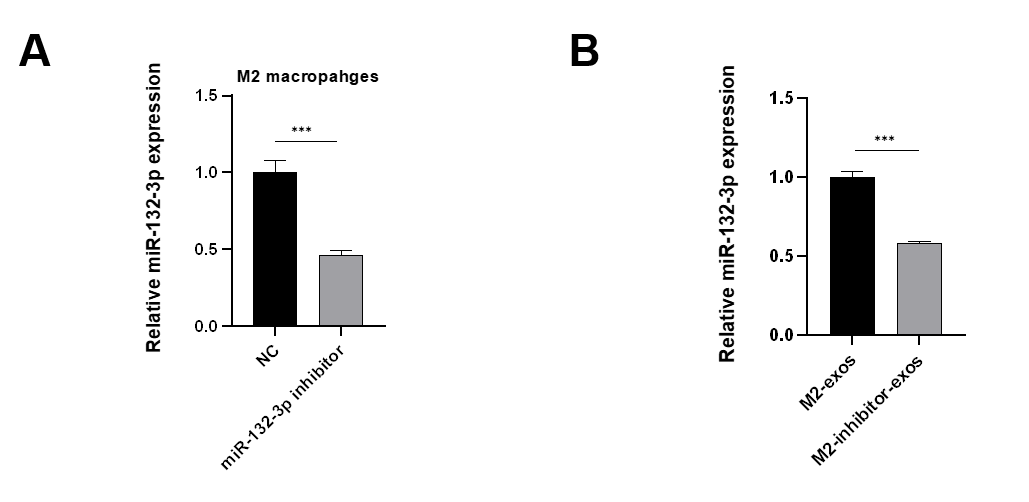


**Figure S6.** miR-132-3p level was detected in M2 macrophages (A) and their derived exosomes (B) after transfected with miR-132-3p inhibitor, ****P*<0.001.


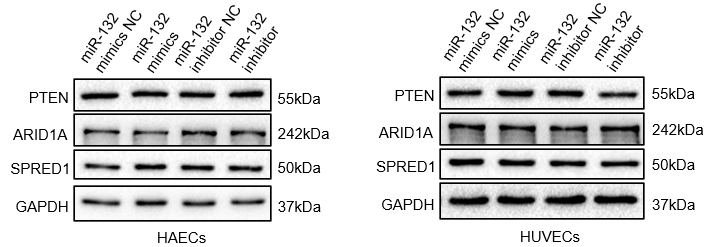


**Figure S7.** Western blot analysis was used to assess the expression of potential target genes after transfecting ECs with miR-132-3p mimics or inhibitors.
